# Thrombotic Thrombocytopenic Purpura Masquerading as Acute Ischemic Stroke

**DOI:** 10.7759/cureus.7661

**Published:** 2020-04-13

**Authors:** Asia Filatov, Emily Kassar, Oladipo Cole

**Affiliations:** 1 Neurology, Charles E. Schmidt College of Medicine, Florida Atlantic University, Boca Raton, USA; 2 Internal Medicine, Edward Via College of Osteopathic Medicine, Auburn, USA; 3 Internal Medicine, Boca Raton Regional Hospital, Boca Raton, USA; 4 Internal Medicine, Florida Atlantic University, Boca Raton, USA

**Keywords:** thrombocytopenic purpura, adamst13, fatrn, stroke

## Abstract

Thrombotic thrombocytopenic purpura (TTP) is a hematologic disorder that results in widespread clotting due to a deficiency of a disintegrin and metalloproteinase with a thrombospondin type 1 motif, member 13 (ADAMTS13) protease. This leads to excessive von Willebrand factor (VWF) protein-platelet multimers. Due to platelet consumption, platelet levels fall, resulting in thrombocytopenia. We examined a case of a 31-year-old female with no significant medical history who presented with expressive aphasia and paresthesias and was sent to the Emergency Department for a stroke workup. Imaging was negative for ischemic or hemorrhagic stroke; however, a complete blood count (CBC) was consistent with anemia and thrombocytopenia, resulting in a high suspicion for TTP. She was admitted to the intensive care unit and given fresh frozen plasma and packed red blood cells. Plasma exchange therapy was initiated, and her aphasia and paresthesias began to improve. The ADAMTS13 level returned at less than 5%, which prompted the initiation of rituximab therapy. During her 36-day hospital stay, she continued to receive daily steroids and plasma exchange and a total of four courses of rituximab. Her platelets steadily climbed, and she was discharged with instructions to follow up with outpatient hematology. TTP is a thrombotic microangiopathy that results in microscopic blood clots anywhere in the body, including the cerebral arteries. This results in the neurologic abnormalities that are often seen in TTP. Because TTP is a rare disease, treatment modalities are still scarce but include steroids, plasma exchange therapy, and rituximab. Novel therapies are on their way, one being caplacizumab, a monoclonal antibody that inhibits VWF from interaction with glycoprotein 1b. A concern highlighted by this case is the exclusion criteria for the administration of tissue plasminogen activator (TPA). As this patient presented with stroke symptoms and a negative head CT, TPA would have been administered had a CBC not returned showing evidence of TTP. This highlights the importance of strict adherence to the American Heart Association/American Stroke Association guidelines that include ensuring that the platelet count is >100,000 prior to the initiation of TPA.

## Introduction

Thrombotic thrombocytopenic purpura (TTP) is a rare hematologic disorder causing widespread clotting, resulting in low platelets. The disease results from a deficiency of the a disintegrin and metalloproteinase with a thrombospondin type 1 motif, member 13 (ADAMTS13) protease, which results in excessive multimers of the von Willebrand factor (VWF) protein-platelet complex attached to the vascular endothelium [1]. The classic pentad known as FATRN (fever, microangiopathic hemolytic anemia [MAHA], thrombocytopenia, renal abnormalities, and neurologic symptoms) represents the disease in its most severe form [2]. Neurologic symptoms are most commonly headache and confusion, but in rare cases it can present as seizures and focal deficits [2]. In the following section, we will examine a patient who presented to her primary care physician with a complaint of upper respiratory symptoms along with nausea, vomiting, diarrhea, and abdominal pain. During her visit, she began to exhibit expressive aphasia and was sent to the Emergency Department (ED) for a stroke workup.

## Case presentation

A 31-year-old female with no significant medical history presented to the ED with garbled speech that began just prior to arrival. In the days prior, she experienced viral upper respiratory symptoms, as well as nausea, vomiting, diarrhea, and fatigue. Of note, she is an elementary school teacher who is frequently exposed to contractible illness. Due to the persistence of her symptoms, she saw her primary care physician. During the visit, she exhibited garbled speech and was sent to the ED, where a stroke workup was initiated. Upon arrival, her altered speech was still present, which included statements such as "I ate Benadryl for breakfast". She also complained of paresthesias.

**Figure 1:**
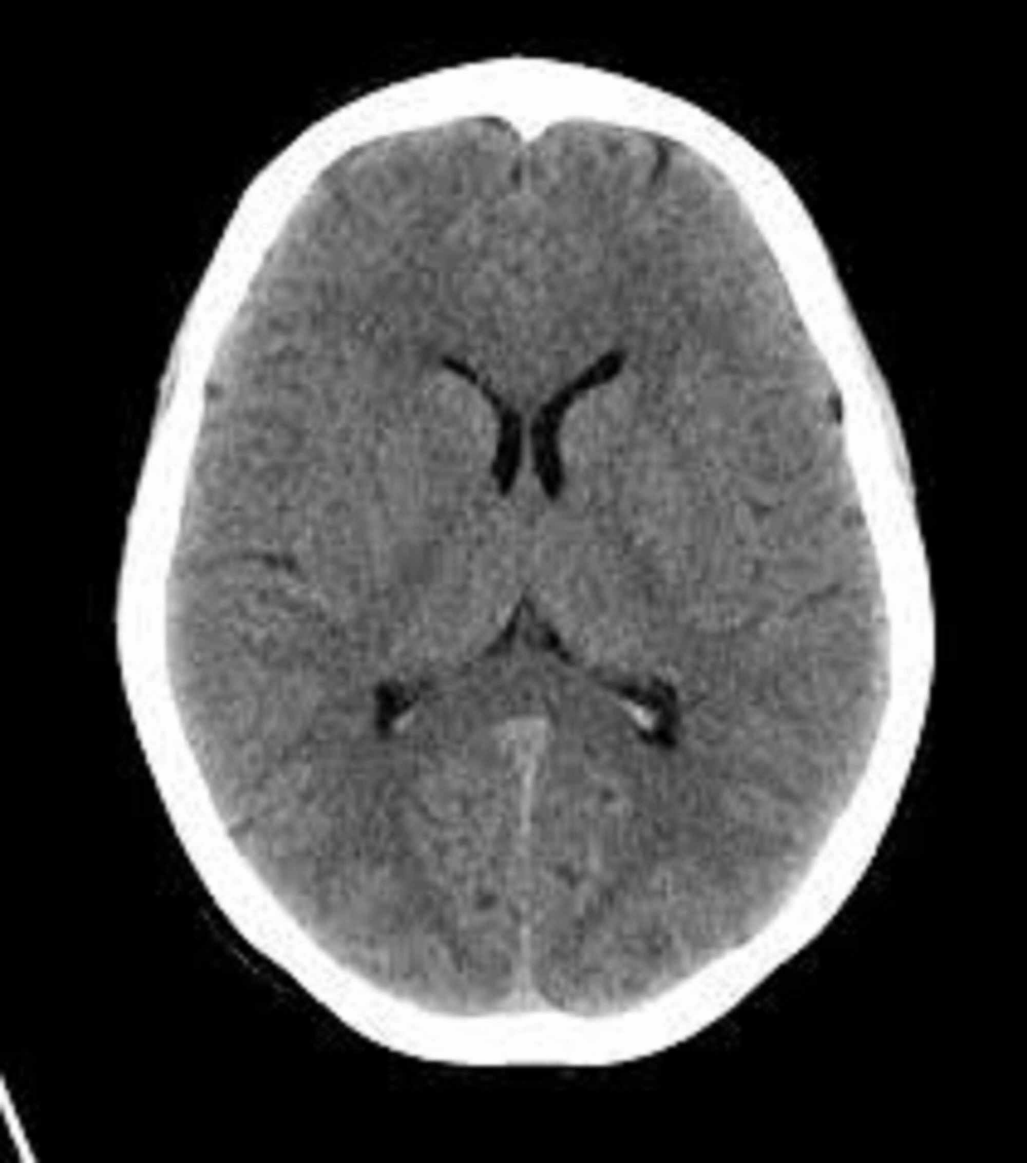
CT of the head: normal findings and no intracranial pathology.

In the ED, the head CT was negative for acute intracranial findings (Figure 1).

She was febrile with a temperature of 38.1 degrees Celsius. The possibility of initiating tissue plasminogen activator (TPA) for acute ischemic stroke was discussed; however, complete blood count (CBC) was returned with a hemoglobin of 6.6, a mean corpuscular volume (MCV) of 93 fL, and a platelet count of 4,000. As the patient had neurologic symptoms with anemia and thrombocytopenia in the setting of viral illness, TTP and hemolytic uremic syndrome (HUS) were included in the differential diagnosis. She was admitted to the intensive care unit and further workup was initiated. A peripheral smear was performed and showed schistocytes. LDH (lactate dehydrogenase) was elevated at 1,154 and haptoglobin was decreased at <8, indicative of intravascular hemolysis [3]. An ADAMTS13 level assay was ordered. Since this test takes several days to return, her PLASMIC score was assessed using several variables including platelet count, MCV, and INR (international normalized ratio) to calculate the probability of severe ADAMTS13 deficiency [4]. Her score was 6, as shown in Table 1, indicating a high risk of severe ADAMTS13 deficiency [4]. 

**Table 1:** Components of PLASMIC score and patient data.

Component	Our patient	Point (0-1)
Platelet count < 30 x 10^9^/L	4 x 10^9^/L	1
Hemolysis (indirect bilirubin > 2 mg/dL, uncorrected reticulocyte count > 2.5%, or undetectable haptoglobin)	Uncorrected reticulocyte 20.4%, haptoglobin > 8	1
No active cancer in the previous year	No active cancer in the previous year	1
No history of solid organ or stem cell transplant	No history of solid organ or stem cell transplant	1
Mean corpuscular volume < 90	Mean corpuscular volume: 93	0
International normalized ratio < 1.5	International normalized ratio: 1.1	1
Creatinine < 2 mg/dL	0.8	1
Total: 6		

**Figure 2:**
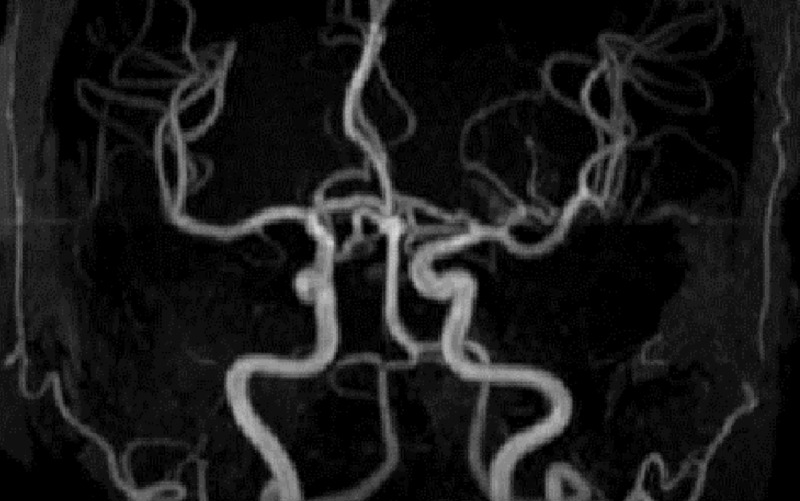
MRA of the brain: normal, negative for an embolic event. MRA, magnetic resonance angiography

A hematologist was consulted. Eight hours after arrival to the ED, she received 4 units of fresh frozen plasma, 2 units of packed red blood cells and two courses of plasmapheresis, resulting in a rapid resolution of aphasia and paresthesias. Her MRA (magnetic resonance angiography) was negative for an embolic event, ruling out ischemic stroke (Figure 2). An infectious workup, which included HIV (human immunodeficiency virus), stool Shiga toxin, mycoplasma, and Epstein-Barr virus, was negative. Her antinuclear antibody was within the normal limits, excluding rheumatologic causes. She was started on steroids and continued to receive daily courses of plasmapheresis. Her ADAMTS13 level returned at less than 5%, confirming TTP, and the decision to start rituximab was made. She received weekly therapy with a total of four treatments during her hospital stay. Plasmapheresis was performed daily, excluding the days that she received rituximab. From a hematologic perspective, she was initially slow to respond. Her platelet count climbed very slowly, with transient dips throughout her hospital stay. The patient continued to be examined by a neurologist and a hematologist. After a 36-day hospital stay, she was discharged with a platelet count of just over 200,000 and a hemoglobin of 10.2. She was placed on daily prednisone and instructed to follow up with outpatient hematology, including weekly bloodwork.



## Discussion

The pathophysiology of TTP is based on the ADAMTS13 protease, which cleaves the VWF multimer. VWF is a protein-platelet complex essential in creating a platelet plug at the endothelium of injured vessels [1]. In the acquired form of the disease, autoantibodies are produced against the ADAMST3 protease, which results in persistent VWF multimers and systemic thromboses. Platelet counts decrease as platelets are consumed causing thrombocytopenia. MAHA in TTP results from shearing forces as red blood cells travel across platelet clumps on the vascular endothelium. MAHA in TTP results from shearing forces as red blood cells travel across platelet clumps on the vascular endothelium, producing fragmented red blood cells known as schistocytes.

Expressive aphasia is an example of neurologic manifestation occurring in TTP resulting from brain damage, particularly the areas concerned with language. Consequently, the patient's speech production gets severely damaged, even though his or her intellect might be intact. The usual cause of aphasia is brain injury or stroke, which damages one or more areas of the brain dealing with language. In most cases, neurologic symptoms including expressive aphasia occur in the course of TTP [3]. This argument is based on the interaction between the circulating platelets and the vascular endothelium that results in a profound dysregulation of coagulation. CT scan of these patients may demonstrate infarcts in specific sections of the brain that correlate with neurologic findings including expressive aphasia [1].

Research studies have demonstrated incidences of atypical TTP in patients with strokes. Idowu and Reddy present a case of a middle-aged woman with atypical thrombocytopenic purpura who presented with an inability to speak, mild dysarthria, and expressive aphasia [5]. The patient showed marked improvement in her neurologic status upon receiving seven TPEs. Crum and O'Brien in their research article reported two TTP cases where initial findings majorly comprised neurologic deficits including expressive aphasia, hence causing delays in diagnosis [6]. These neurologic deficits were later followed by hematologic manifestations. A research study by Boattini and Procaccianti has also shown that TTP is commonly associated with abnormal brain neuroimaging and that therapeutic plasma exchange (PEX) is useful in resolving the symptoms [7]. A case report by Azmi and Maizuliana of a 38-year-old lady who presented with TTP, expressive aphasia, and right-sided body weakness also shows that these symptoms may commonly occur together [8].TTP is a hematologic condition that is described by a pentad of neurologic and renal abnormalities, fever, anemia, and thrombocytopenia. Patients with TTP in most cases present with neurologic deficits; however, expressive aphasia is rare, hence requiring further investigations to ensure effective management.

The diagnostic test for TTP is an ADAMTS13 assay, which expresses ADAMTS13 activity as a fraction of normal activity within pooled plasma [9]. Given that ADAMTS13 assay takes several days to return, a preliminary diagnosis can be made clinically if the patient presents with MAHA and thrombocytopenia without another known cause and without acute renal failure. If acute renal failure is present, HUS is more likely. A PLASMIC score, as was calculated in our patient, is often used to determine the probability that severe ADAMTS13 protease deficiency is present based on several clinical findings, with 1 point assigned to each. In 2018, a study evaluated the success of PEX therapy in patients with low intermediate (0-5) risk scores and high (6-7) risk scores [4]. In the high-risk group, treatment with PEX therapy leads to significantly increased survival [4]. With a PLASMIC score of 6, the present patient is considered at high risk, and PEX was conducted prior to the return of an ADAMTS13 level assay.

TTP is a recognized rare disease according to the National Heart, Lung, and Blood Institute, a center part of the National Institutes of Health. As with many rare diseases, treatment modalities are scarce, but novel studies are underway [10]. In the subsequent paragraphs, the accepted treatment guidelines are reviewed, in addition to other treatment modalities used in refractory cases as well as novel biologics being studied currently.

The recognized guideline treatment for TTP is plasmapheresis or PEX for acquired TTP or plasma therapy for inherited TTP. For inherited TTP, plasma therapy is instituted to replace the ADAMTS13 enzyme [7,10-13]. In acquired TTP, PEX is used to remove anti-ADAMTS13 enzyme antibody and to replete the enzyme itself. A once fatal process now has a survival benefit of up to 85% using PEX [12,13]. As soon as a diagnosis is made or suspect TTP, PEX should be initiated at 1.5x plasma volume exchange for the first procedure and 1.0x plasma volume exchange for subsequent treatments. This process is performed until platelet concentration reaches normal levels, organ involvement has resolved, and hemolysis has terminated [8,13].

Steroids are often the mainstay of treating autoimmune disease such as immune thrombocytopenia, systemic lupus erythematosus, and Sjogren’s syndrome to name a few. However, evidence of its effectiveness in treating TTP is minimal at best. Coppo and French Reference Center for Thrombotic Microangiopathies evaluated the outcomes in various studies and concluded that steroids given in combination with PEX versus PEX alone were equivalent [11-13]. Furthermore, it has been observed that relapse occurs more frequently with steroid treatment. Nevertheless, current guidelines recommend the initiation of systemic steroids of 1.5 mg/kg/day for three weeks, which is quite reasonable [12]. Some studies even suggest using high-dose methylprednisolone (10 mg/kg/day for three days followed by 2.5 mg/kg/day) as an adjunctive treatment with PEX for patients with new-onset TTP, achieving a modest 78% remission after 23 days of treatment in a small study [13].

Rituximab is an anti-CD20 monoclonal antibody originally developed to treat B cell malignancies [7,13]. However, several trials have shown its effective properties and high response to treat TTP, and it has now become the mainstay first-line treatment in both the acute phase and refractory cases (i.e., exacerbations or no improvement in clinical features or lack of platelet response within four days of PEX) and has led to remission in one to four weeks in patients [7,11-13]. For acute cases, two prospective trials resulted in shorter hospitalization and fewer relapses in patients who did not respond optimally to PEX. As with other treatments discussed below, targeting the inhibition in the production of anti-ADAMTS13 antibody with four infusions at 375 mg/m2/week after PEX is the goal using rituximab. This therapy quickly reduces peripheral antibody-producing B cells, thus causing a rapid and substantial reduction in anti-ADAMTS13 antibody. Remissions have been significant within the first year of therapy. These are considerably significant when compared with other modalities of therapies.

Cyclosporine and vincristine have historically been used in refractory TTP. Both of these agents have been reported to have a steady remission rate in refractory TTP, up to 73%, and in some instances are being used as frontline agents in a small subset of patients, when response to PEX is inadequate. Nevertheless, the literature and specialists consider these choices as secondary or salvage therapy after rituximab [8,13]. These two agents have suppressive properties of the anti-ADAMTS13 antibody. However, novel randomized studies have resulted in a more significant response, with steroids decreasing the serum concentration of anti-ADAMTS13 antibody, questioning these two agents’ utility in treatment.

Rare diseases such as TTP are on the path of achieving medical breakthroughs with novel antigen/antibody-targeted treatment modalities. caplacizumab, an inhibitor of VWF-glycoprotein 1b interaction (formerly ALX-0081), may pave the way for the HERCULES trial, which is a phase III double-blind placebo-controlled study which is using caplacizumab to show promising results. Significant results such as shorter time to platelet recovery, decrease in ischemic organ dysfunction through inflammatory biomarkers, and reduction in the incidence of exacerbations are paving the way to make this drug quite promising.

One concern highlighted by this case is the exclusion criteria for TPA administration in a patient presenting with neurologic deficits and a negative head CT. According to the American Heart Association (AHA) and the American Stroke Association (ASA) guidelines, the inclusion criteria for initiation of TPA includes a clinical diagnosis of ischemic stroke with neurologic deficit, time from symptom onset between 3 and 4.5 hours, and no absolute contraindications [14]. As per the ASA/AHA, a platelet count of <100,000 is an absolute contraindication, and a CBC is required as part of immediate testing in a patient with stroke symptoms [14].

A retrospective cohort study published in 2015 examined the importance of awaiting the results of a CBC prior to initiating TPA. The study population consisted of patients receiving TPA in a hospital in both China and the United States and showed that a significantly shorter door-to-needle interval was found in a group of patients who received TPA prior to the return of CBC results [13]. Additionally, of the patients who received TPA before CBC was returned, 98.8% of patients had normal results [13]. The remaining patient had a platelet count of 88,000, and no adverse event occurred from TPA administration. Applying the results of this study would have been catastrophic in our patient and raises several pertinent questions: if time is of the essence and we cannot wait for a CBC to return, how often would we be causing a negative outcome, most importantly, intracranial hemorrhage? In a patient with no risk factors for ischemic stroke, including young age and no significant medical history, should we be concerned for a hematologic cause and wait for a CBC even if we risk closing the window for TPA administration?

## Conclusions

TTP is an autoimmune condition that affects the coagulation system, resulting in the formation of microscopic blood clots in any blood vessel such as the cerebral arteries. Clinical presentation of TTP occurs due to end-organ damage and reduced blood flow. Neurologic manifestations such as expressive aphasia, altered mental status, visual disturbances, seizures, and paresthesia can often be experienced by these patients as a result of cerebral ischemia caused by microscopic clots occurring in the cerebral arteries. Patients with TTP can present to the emergency setting in the same manner as patients with an acute stroke with negative CT imaging. This can prompt physicians to treat these patients as ischemic stroke patients and administer TPA, which highlights the importance of checking a CBC prior to stroke intervention.
